# Targeting autophagy to sensitive glioma to temozolomide treatment

**DOI:** 10.1186/s13046-016-0303-5

**Published:** 2016-02-02

**Authors:** Yuanliang Yan, Zhijie Xu, Shuang Dai, Long Qian, Lunquan Sun, Zhicheng Gong

**Affiliations:** Department of Pharmacy, Xiangya Hospital, Central South University, Changsha, 410008 China; Institute of Hospital Pharmacy, Central South University, Changsha, 410008 China; Department of Pathology, Xiangya Hospital, Central South University, Changsha, 410008 China; Center for Molecular Medicine, Xiangya Hospital, Key Laboratory of Molecular Radiation Oncology of Hunan Province, Central South University, Changsha, 410008 China

**Keywords:** glioblastoma, temozolomide, treatment resistance, autophagy

## Abstract

Temozolomide (TMZ), an alkylating agent, is widely used for treating primary and recurrent high-grade gliomas. However, the efficacy of TMZ is often limited by the development of resistance. Recently, studies have found that TMZ treatment could induce autophagy, which contributes to therapy resistance in glioma. To enhance the benefit of TMZ in the treatment of glioblastomas, effective combination strategies are needed to sensitize glioblastoma cells to TMZ. In this regard, as autophagy could promote cell survival or autophagic cell death, modulating autophagy using a pharmacological inhibitor, such as chloroquine, or an inducer, such as rapamycin, has received considerably more attention. To understand the effectiveness of regulating autophagy in glioblastoma treatment, this review summarizes reports on glioblastoma treatments with TMZ and autophagic modulators from in vitro and in vivo studies, as well as clinical trials. Additionally, we discuss the possibility of using autophagy regulatory compounds that can sensitive TMZ treatment as a chemotherapy for glioma treatment.

## Background

Glioblastoma or glioblastoma multiform (GBM) is an aggressive astrocytic cell neoplasm and one of the leading causes of cancer-related deaths in both pediatric and adult populations. The median survival of patients with GBM is approximately 12–15 months after the initial diagnosis. Conventional therapies for patients with newly diagnosed GBM include surgical tumor resection followed by radiation therapy and chemotherapy. Though these therapeutic methods have increased the survival rate to 14.6 months, the survival advantages are only palliative [1, 2]. In March 2005, the U.S. Food and Drug Administration approved temozolomide (TMZ) concomitantly with radiotherapy for the treatment of adults with newly diagnosed glioblastoma as well as using TMZ alone as a maintenance treatment [3]. To date, TMZ is the most widely used and effective first-line chemotherapeutic drug for glioblastoma patients [4, 5], although several chemotherapeutic agents can be found on the current pharmaceutical market [3].

Autophagy is activated in tumor cells by chemotherapeutic agents and radiation [6, 7], and the process constitutes a potential target for cancer therapy. Since autophagy was discovered, it has been thought to act as a pro-survival or pro-death response to several stresses, especially chemotherapy and radiotherapy, at the cellular and organic levels [8]. The mechanism by which autophagy could perform these seemingly opposite roles remained elusive until recently. Under moderate stimulus conditions, the autophagic pathway operates to supply cells with metabolic substrate, contributing to the maintenance of cell survive [9]. However, a considerable body of literature reports that uncontrolled autophagy is also a cell death mechanism that can occur either in the absence of detectable signs of apoptosis or concomitantly with apoptosis [10]. Similarly, using C. elegans as a model system, Kang C et al. found that physiological levels of autophagy promote optimal survival of C. elegans upon stresses, whereas either insufficient or excessive levels of autophagy are pro-death [11]. In addition, for a multicellular organism, autophagic cell death might well represent another pro-survival mechanism, which provides metabolic supplies during whole-body nutrient deprivation via the heterophagy [9].

As the dual roles of autophagy in the response to chemoradiotherapy, the modulation of autophagy in response to therapeutics could have anti-cancer efficacy as well as help with therapy resistance [6]. In some cases, autophagy-delayed apoptotic death (type I programmed cell-death) in cancer cells undergoing therapeutic treatment; the treatment of these cells with autophagy inhibitors, such as chloroquine (CQ); or the knockdown of autophagy genes, including Beclin1 and other ATG genes, enhanced therapy-induced apoptosis [12]. Autophagy also contributes to promote cell survival [13], and blocking the autophagic process increases the efficacy of a variety of anti-cancer agents [14]. However, according to other studies, various therapeutic methods could enhance autophagic cell death (type II programmed cell-death) in glioblastomas [15], hepatocellular carcinoma [16], etc. Thus, the potential clinical applications for the monitoring of autophagy in gliomas and other cancers require the detection of current therapeutic effects and the development of novel anticancer strategies. These treatment strategies include the induction of autophagy to enhance its antitumor effects and the inhibition of autophagy to induce apoptosis [17].

In GBM, TMZ-induced autophagy is putative mechanism of TMZ action in cancer cells and patients [6, 18]. It has been proposed that autophagy could lead to either cancer cell survival or cell death, depending on the cellular context [19, 20]. On one hand, TMZ-induced autophagy seems to have a cytoprotective role. Lenz G’s group demonstrated that acute treatment with TMZ induces the sustained inhibition of Akt-mTOR (the mechanistic target of rapamycin (serine/threonine kinase)), which produced a transient induction of autophagy, leading to cell resistance of the therapy [21]. On the other hand, Gao S et al. found that the cytotoxicity of TMZ to glioma cells was enhanced by autophagy when combined with thalidomide, a drug proposed to affect the PI3K (phosphatidyl inositol 3 kinase) /Akt/mTOR pathway, which plays a role in autophagy regulation [22]. Accordingly, autophagic cell death was found to be necessary for the antitumor effects of the combination of TMZ and radiotherapy [23]. These data are compatible with the theory that autophagy is mostly a survival process, whereas mortal autophagic flux most easily achieved by a combination treatment can be exploited in anticancer therapy. To understand the effectiveness of regulating autophagy in glioblastoma treatment, this review focuses on reports on glioblastomas treated with TMZ and autophagic modulators from in vitro and in vivo studies, as well as clinical trials.

## Known resistance mechanisms of TMZ

**Fig. 1:**
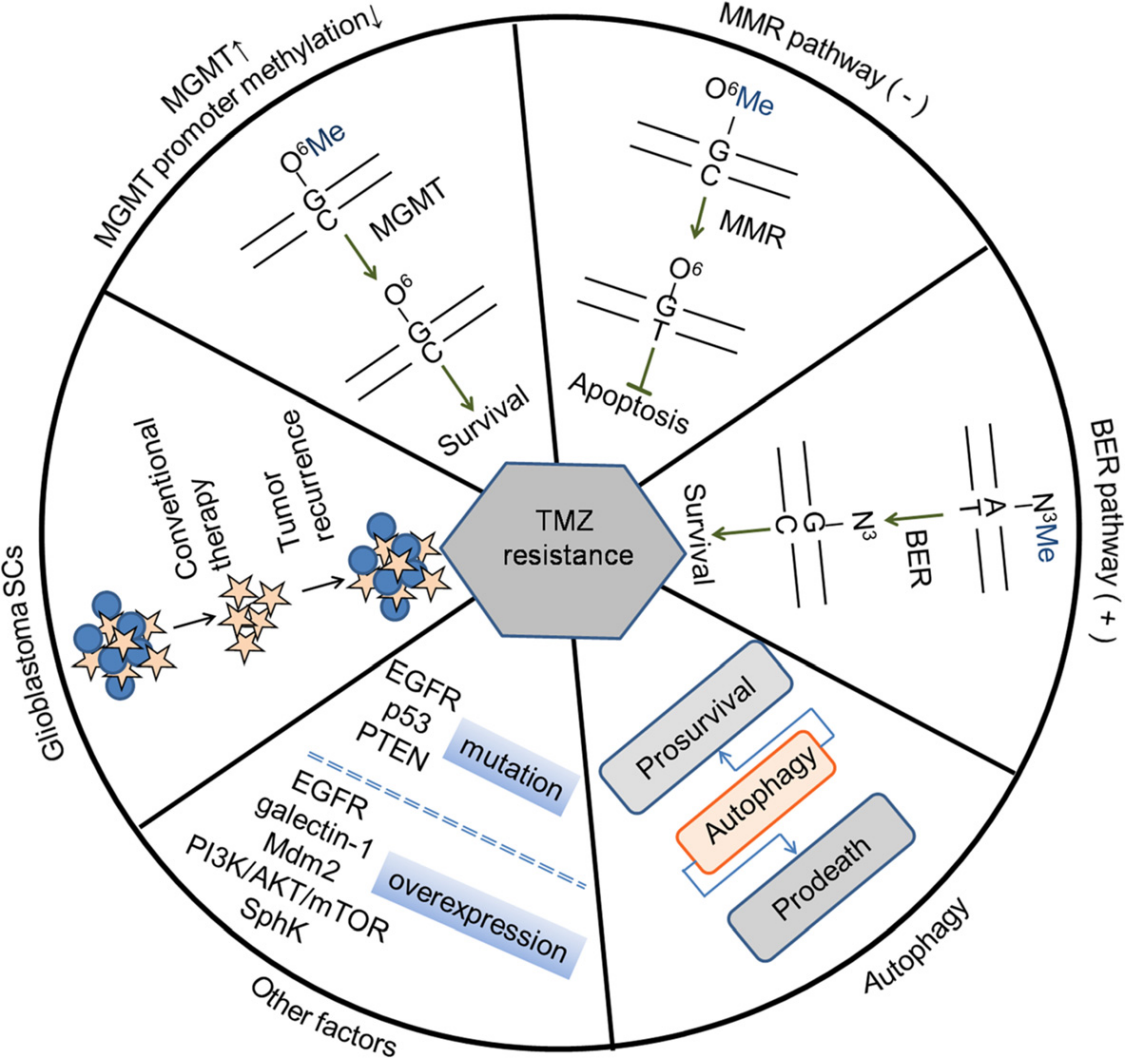
The known resistance mechanisms of TMZ in glioblastoma treatment

TMZ is a small lipophilic molecule (194 Da) and an orally available imidazotetrazine-class alkylating agent [24]. The cytotoxicity of TMZ is thought to result from the formation of O^6^-methylguanine (O^6^MeG) in DNA, which mispairs with thymine during DNA replication, triggering futile cycles of the mismatch repair system and resulting in subsequent DNA damage [25]. Due to its ease of administration, tolerability, and known capacity to cross the blood–brain barrier, TMZ provides modest antitumor activity and is currently used to treat glioblastomas. In addition, fibrin glue (FG), a drug delivery system, can effectively administer TMZ directly to the target tumor and exert antitumor effects, with no severe damage to the normal brain tissue [26]. TMZ is considered the most effective drug for the treatment of GBM. However, overtime, GBM cells become resistant to the cytotoxicity caused by TMZ. This resistance is related to the implementation of several mechanisms, discussed below (Fig 1).

One of the well-documented mechanisms in GBM resistance involves O^6^MeG DNA methyltransferase (MGMT). MGMT can eliminate the TMZ-induced DNA damage by removing the methyl group in the O-6 position of the substrate guanine, further generating TMZ resistance [27]. Additionally, as the level of MGMT is inversely related to the density of cytosine phosphate guanine (CpG) methylation in CpG islands, the decreased methylation of the MGMT promoter improves survival after TMZ chemotherapy [28]. To date, therapeutic molecules inhibiting MGMT, such as O^6^-(4-bromothenyl) guanine and O^6^-benzyl guanine, have been used in clinical trials preceding treatment with TMZ.

DNA mismatch repair (MMR) [29] and base excision repair (BER) [30] are the primary DNA repair systems involved in TMZ resistance mechanisms. The MMR system can correct nucleotide base mismatches generated during DNA synthesis. When MGMT is reduced or absent, the existence of O^6^MeG can be recognized by MMR protein complexes and paired with thymine to form O^6^MeG/T. The futile cycles of the insertion and excision of thymine mentioned above lead to cell cycle arrest and apoptosis. Conversely, the impairment of the MMR pathway causes a failure in the ability to recognize O^6^MeG/T, resulting in less effective TMZ treatment. The BER system participates in the repair of DNA damage caused by alkylating agents. TMZ is thought to form N3 and N7 methylations in DNA, which is lethal if not repaired. BER can repair N3 lesions, giving rise to a TMZ resistant phenotype.

**Fig. 2:**
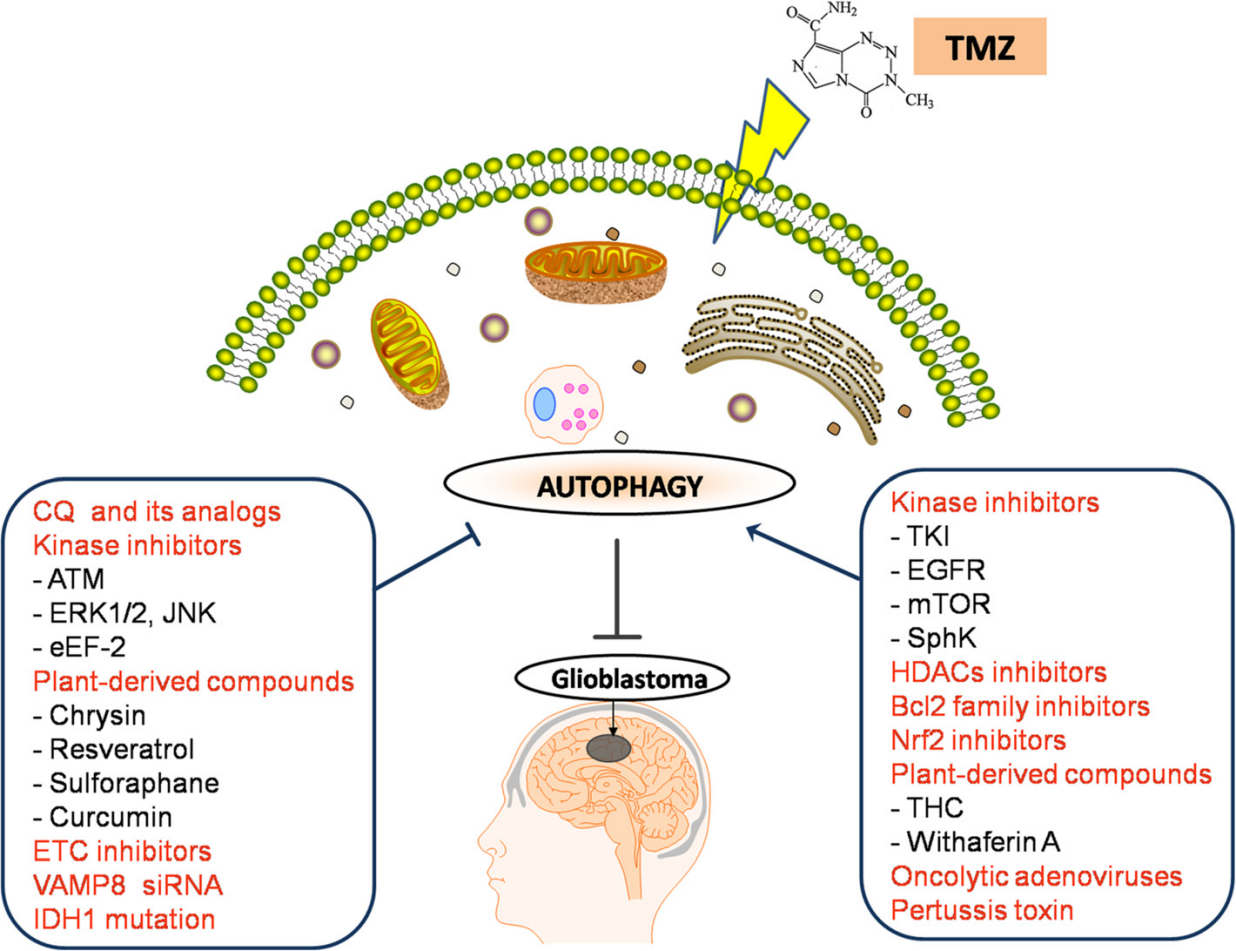
Effect of autophagy modulation on the TMZ anti-glioblastoma activity

Apart from DNA repair systems, it has been demonstrated that other factors, including epidermal growth factor receptor (EGFR) [31], phosphatase and tensin homolog (PTEN) [32], galectin-1 [33], murine double minute 2 (Mdm2) [34], p53 [35], PI3K/AKT/mTOR pathway [36], and sphingosine-1-phosphate/ sphingosine kinases [37], play important roles in TMZ resistance. In addition, another recent study showed TMZ-resistant glioblastoma stem cells (GSCs) had enriched MGMT promoter methylation, suggesting intrinsic or rapidly acquired resistance, in which the details of the specific mechanism are still unclear [38]. Although the above mechanisms by which GBM cells become resistant to anticancer drugs has been elucidated, autophagy, an important evolutionarily conserved catabolic process, is now emerging as a crucial player in TMZ resistance. Autophagy can be viewed as having a controversial pro-death or pro-survival role in response to TMZ treatment (Fig 2).

## Effect of autophagy modulation on the TMZ anti-glioblastoma activity

### Autophagy as a cytoprotective role

TMZ is considered the most effective drug in the treatment for GBM. However, its efficacy is often limited by tumor recurrence and the development of resistance to TMZ. Autophagy, upon TMZ treatment, mostly functions as a survival mechanism, as its inhibition greatly ameliorates the level of apoptosis following TMZ treatment at therapeutically relevant doses (≦100 μM), suggesting that the inhibition of autophagy may ameliorate the therapeutic outcome of TMZ-based cancer therapy [39, 40] (Tables 1 and 2). Therapeutic molecules inhibiting autophagy, such as CQ and its analogs, have been used in clinical trials preceding treatment with TMZ [41]. Furthermore, the autophagy suppressing functions of Ataxia-telangiectasia mutated (ATM) kinase inhibitors and plant-derived compounds, such as resveratrol, have been demonstrated, including the reduction of tumor volumes and the prolonged survival in mouse xenograft [42, 43]. Autophagy is the process in which damaged or unwanted cytoplasmic constituents are segregated into autophagosomes and designated for lysosomal degradation. Autophagy has a cytoprotective role mainly through eliminating intracellular pathogens via the activation of the innate and adaptive immune responses, and it is also noted for its role in maintaining endoplasmic reticulum (ER) and metabolic homeostasis in tumor cells undergoing chronic hypoxia and nutrient depletion.

**Table 1:** In vitro studies of autophagy inhibiors on the TMZ anti-glioblastoma activity

Cell lines	Therapeutic methods (concentration /exprosure time)	Major findings	Interpretation	Reference
Rat C6 cells	TMZ 100–1,000 μM/24 hours	CQ potentiated TMZ-induced cytotoxicity.	CQ increaseed cellular ROS in glioma cells by inhibiting mitochondrial autophagy.	[35]
Human U87 cells	CQ 10 μM/24 hours			
Human LN229, U251 and U87 cells	TMZ 20–100 μM/48 hours	CQ increased the chemosensitivity of glioma cells to TMZ.	CQ blocked autophagy and triggered endoplasmic reticulum stress.	[48]
	CQ 10-25 μM/48 hours			
Human GBM8901cells	TMZ 100 μM/24 hours	Chrysin induced apoptosis, suppressed migration and invasion, and sensitized GBM cells to TMZ.	Chrysin inhibited TMZ-induced autophagy and MGMT expression.	[75]
	Chrysin 20 μM/24 hours			
Human U87, GBM8401 and GBM-SKH cells	TMZ 400 μM/72 hours	Resveratrol enhanced the therapeutic effect of TMZ against malignant glioma.	Coadministration of resveratrol and TMZ reduced tumor volumes by suppressing ROS/ERK-mediated autophagy.	[42]
	Resveratrol 10 μM/1 hours			
Human U87, U251 and SHG‑44U87 cells	TMZ 100 μM/72 hours	ATM inhibitor ku-55933 enhanced TMZ cytotoxicity in inherently TMZ‑sensitive glioma cells.	Ku-55933 inhibited the phosphorylation of AMPK, and reduced the levels of TMZ-induced autophagy.	[54]
	Ku-55933 10 μM/72 hours			
Human U87 and U251 cells	TMZ 100 μM/72 hours	TMZ chemoresistance was overwhelmed by targeting ATM.	Ku‑55933 inhibited the activation of ULK1 and interrupted the cytoprotective process of autophagy.	[55]
	Ku-55933 10 μM/72 hours			
Human U-118 cells	TMZ 0–500 μM/24-48 hours	Inhibition of ERK1/2 partially eradicated the chemoresistance of U-118 GBM cells to TMZ.	ERK1/2 specific inhibitors U-0126 prevented the activation of autophagy by TMZ.	[19]
	U-0126 15 mM/48 hours			
Human U87 cells	TMZ 400 μM/0–72 hours SP600125 10 μM/1 hours	TMZ-induced autophagy is mediated by JNK activation.	JNK inhibitor suppressed TMZ-induced JNK phosphorylation, further blocked autophagy and increased apoptosis.	[61]
Human LN229 and U251 cells	TMZ 100 μM/24 hours	Targeting eEF-2 kinase can enhance the anti-glioma activity of TMZ.	Inhibition of eEF-2 kinase by SiRNA or NH125 blocked the activation of TMZ-induced autophagy.	[65]
	eEF-2 SiRNA N/A			
	NH125 0.5 μM/24 hours			
Human U251 cells	TMZ 200 and 400 μM/72 hours	Inhibition of autophagy potentiated the cytotoxicity of curcumin or TMZ as well as TMZ/curcumin combination.	Autophagy inhibition sensitizes TMZ and curcumin treated cells to apoptosis.	[81]
	Curcumin 15 μM/72 hours			
	3-MA 4 mM/72 hours			
Human U87 cells	TMZ 400 μM/36-72 hours	TMZ induced autophagy through mitochondrial damage- and ER stress-dependent mechanisms to protect glioma cells.	ETC inhibitors rotenone, sodium azide, oligomycin, or ER stress inhibitor 4-PBA reduced autophagy and consequently increased TMZ-induced apoptosis.	[61]
	rotenone 20 nM/1 hour			
	sodium azide 150 μM/1 hour			
	oligomycin 1 nM/1 hour			
	4-PBA 10mM/1 hour			
Human U87 and U251 cells	TMZ 50–200 μM/48 hours	Targeting VAMP8 alleviated TMZ resistance in glioma cells.	silencing of VAMP8 by SiRNA could impaire the TMZ-induced autophagic flux.	[69]
	VAMP8 SiRNA N/A

**Table 2:** In vivo studies of autophagy inhibitors and inducers on the TMZ anti-glioblastoma activity

Effect of autophagy modulation	Subject	Agent regimen	Major findings	Interpretation	Reference
cytoprotective role	Xenografts of human U87 MG GBM cells in male athymic nu/nu mice	CQ 10 mg/kg + TMZ 5 mg/kg given by oral gavage for 48 hours with water.	CQ enhances the cytotoxic effects of TMZ by blocking autophagy.	CQ in combination with TMZ significantly increased the amounts of LC3B-II, CHOP/GADD-153, and cleaved PARP.	[48]
	Xenografts of human U87 GBM cells in athymic nude mice	50 mg/kg of QNX for 24 hours; 25 mg/kg QN, CQ, MFQ, or QNX for 48 hours.	QBAs, a novel class of autophagy inhibitors, are holding the promise for the coadministration treatment of gliomas.	QNX selectively accumulates in tumor cell vacuoles. QBAs have the ability to induce ER stress potentially leading to apoptosis.	[49]
	Xenografts of human U87 MG GBM cells in nude mice	Resveratrol 12.5 mg/kg + TMZ 10 mg/kg injected intraperitoneally for 12 days.	Resveratrol increases the effect of TMZ in glioma xenografts by reduceing tumor volumes.	Coadministration of resveratrol and TMZ suppressing ROS/ERK-mediated autophagy and subsequently inducing apoptosis	[42]
	Xenografts of SJG2 pediatric GBM in NOD-SCID mice	MA 100 mg/kg + TMZ 65 mg/kg given by oral gavage for two weeks.	Combination had a significant increase in survival.	ATM-MPG axis will lead to improved treatment of alkylating agent-resistant tumors.	[43]
Autophagy-associated cell death	Xenografts of human U87 and T98 GBM cells in nude mice	THC 15 mg/kg + TMZ 5 mg/kg injected peritumorally for 14 days in 100 mL of PBS supplemented with 5 mg/mL defatted and dialyzed BSA.	Combined treatment with THC and TMZ strongly reduces the growth of glioma xenografts.	Combined treatment with THC and TMZ enhances autophagy-mediated cell death.	[86]
	Xenografts of human U87 MG GBM cells in female BALB/c nu/nu mice	AdWT or CRAd-S-pk7 3×10^9^ vp in 5 μl + TMZ 70 or 10 mg/kg in 100 μl injected with five consecutive intraperitoneal.	In which 90% of the mice with intracranial tumours were long-term survivors after treatment with TMZ and CRAd-S-pk7.	As both LC3 and cleaved Caspase-3 expressed, both autophagy and apoptosis are responsible for cell death.	[90]

## Chloroquine and its analogs

CQ and its close quinoline-based analogues were developed primarily to treat malaria [44]. However, they are increasingly recognized for their effectiveness in a myriad of non-malarial diseases [45].They have been shown to have antagonistic effects in late autophagy by controlling acidic lysosomes, and they currently have established roles in the treatment of different cancers, including glioblastoma [46]. With the aim of determining the molecular mechanisms of enhancing the chemotherapeutic effect of CQ on malignant glioblastomas, recent studies have been dedicated to probing the cytotoxicity of CQ combined with TMZ. Reactive oxygen species (ROS) are one of the main causes of dysfunctional or damaged mitochondria. Hori YS et al. found that CQ increases cellular ROS and augments TMZ cytotoxicity in glioma cells by inhibiting mitochondrial autophagy. The knockdown of Beclin1 by siRNA mimics the ROS-mediated enhancement of cell death induced by CQ [47]. TMZ treatment results in G2/M phase arrest, although apoptosis occurs in a few of the treated glioma cells. CQ could potentiate the chemosensitivity of glioma cells to TMZ via blocking autophagy, which is dependent on the status of p53 [35]. TMZ combined with CQ synergistically inhibits cell growth through G2/M arrest in glioma cells expressing mutant p53, while in cells expressing wild type p53, the combination therapy induces cell death via apoptosis. A previous study established that CQ enhances TMZ cytotoxicity in gliomas by blocking the ER chaperone and cell survival protein GRP78/BiP-dependent autophagy and inducing the expression of CHOP/GADD-153 (an ER stress proapoptotic protein) and PARP (an apoptotic maker) [48]. Furthermore, similar results have been obtained for other quinoline-based antimalarial (QBA) drugs, such as hydroxychloroquine (HCQ), quinine (QN), mefloquine (MFQ), and quinacrine (QNX). The order of the inhibition potency of QBAs on autophagy is as follows: QNX > MFQ > HCQ > CQ > QN. In addition, the antitumor activity of the most potent compound, QNX, could selectively accumulate in tumor cell vacuoles in vivo [49].

Targeting autophagy by CQ has attracted attention as an adjuvant therapy for glioblastoma patients (ClinicalTrials. gov Identifier: NCT00486603, NCT02378532, NCT01430351. http://clinicaltrials.gov) (Table 3), because Briceño E et al. reported the efficacy of adding CQ to conventional treatment in a prospective controlled randomized trial [50]. As CQ has a strong antimutagenic effect and a good toxicological profile, the chronic administration of CQ greatly enhanced the response of GBM to antineoplastic treatment [50, 51]. Moreover, the combination of the CQ-analogue HCQ and TMZ significantly increases the number of therapy-associated autophagic vacuoles in the peripheral blood mononuclear cells of glioblastoma patients but with a dose-limiting toxicity [18]. Taken together, these reports suggest that CQ is likely to be beneficial for the treatment of gliomas and holds promise as an effective adjuvant therapy in glioma patients. However, with some limitations, such as differences in pretreatment characteristics and conventional treatment regimens, larger trials are warranted to further confirm the benefit of CQ and CQ-analogs.

**Table 3:** Clinical trials of CQ-analogs combined with TMZ in cancer therapy

Studys	Type	Year of registration	Sponsor	Drugs	Tumor type	Targeted enrolment	Status	Major findings
NCT00486603 [41]	Phase I	2007	Sidney Kimmel Comprehensive Cancer Center	200, 400, 800 mg/day HCQ + 150-200 mg/m2/day TMZ for 5 d [q4wk] + RT	Newly diagnosed GBM	16	Complished	HCQ 600 mg/day was found to be the MTD in this combination.
NCT00486603 [41]	Phase II	2007	Sidney Kimmel Comprehensive Cancer Center	600 mg/day HCQ + 150-200 mg/m2/day TMZ for 5 d [q4wk] + RT	Newly diagnosed GBM	76	Complished	Median survival of 15.6 mos with survival rates at 12, 18, and 24 mo of 70%, 36%, and 25%. PK analysis indicated dose-proportional exposure for hCQ. AV in PBMC: patients with Cmax above 1785 ng/mL had a increased median AV change of 1.01.
NCT02378532^a^	Phase I	2015	Maastricht Radiation Oncology	200-600 mg/day HCQ + 150-200 mg/m2/day TMZ for 5 d [q4wk] + RT	Newly diagnosed GBM	9	Not yet open	N/A
NCT01430351^a^	Phase I	2011	M.D. Anderson Cancer Center	250 mg/day MFQ for 3 d/week+ 150 mg/m2/day TMZ for 5 d [q4wk]	Post-RT Glioblastoma	144	Recruiting participants	N/A

^a^ Further information can be found at http://clinicaltrials.gov

As the anti-tumor mechanism of CQ, autophagy inhibition has attracted much more attention to potentiate TMZ cytotoxicity. The combination of TMZ and CQ-analogs is an efficient alternative strategy in glioma treatment and could improve with clinical development. The application of an optimal dose of CQ and TMZ and the treatment schedule are important for the synergistic effect of the combination of the drugs. However, the detailed mechanistic role of CQ-analogs and their derivations as an enhancer of TMZ needs to be further examined.

## Kinase inhibitors

A recent study has found through database mining and mutation analysis, that approximately 34 kinase genes are mutationally activated at considerable frequencies in glioblastomas [52], indicating that kinase inhibition studies could offer new opportunities for the rational development of therapeutic approaches for glioblastomas.

ATM kinase forms a central node in the DNA damage response phosphorylation cascade by contributing to the initiation, amplification and transmission of the DNA damage signal to downstream substrates [53]. A study by Wang W’s group demonstrated that, in glioblastomas, TMZ treatment induces cytoprotective autophagy through the ATM-AMPK (adenosine monophosphate-activated protein kinase) pathways, which occurs in a MMR protein-MLH1-dependent manner. The ATM inhibitor ku-55933 can abrogate the ATM-AMPK signaling pathway, which further enhances TMZ cytotoxity in glioma cells [54]. Meanwhile, the interruption of the ATM-AMPK pathways ku-55933 inhibits the cytoprotective process of autophagy, which results in the augmentation of the TMZ cytotoxic effect and promotes glioma cell death under apoptotic stress [55]. In addition, Nadkarni A et al. found the inhibition of ATM activation by ku-55933 suppresses the repair of TMZ-induced DSBs (DNA double-stranded breaks) in inherently TMZ-sensitive tumor lines [56]. The loss of ATM-mediated BER results in increased alkylating agent-induced cytotoxicity in vitro and prolonged survival in vivo [43]. These results suggest that ku-55933 may be an effective TMZ-sensitizing agent.

The mitogen-activated protein kinase (MAPK) signaling pathway is usual activated by upstream genomic events and functions as a tumor suppressor and more commonly, a pro-oncogenic signal [57]. Therapies targeted toward MAPK/extracellular signal-regulated kinase (ERK) components have various response rates when used in different solid tumors, such as glioblastomas [58]. Previous studies have reported that in patient tumor tissue samples, ERK was phosphorylated, indicating that this survival pathway was active in glioma cells [59]. Lin CJ et al. revealed that TMZ induces the generation of ROS and the activation of ERK, which consequently leads to protective autophagy in glioma cells [42]. Because ERK signaling pathways sustain key features that characterize gliomas, i.e., enhanced proliferation and invasion, protection from proapoptotic stimuli and the activation of autophagy, it is likely that they may contribute to TMZ chemoresistance. The results from Lopes MC’s group demonstrated that the chemoresistance of U-118 GBM cells to TMZ was partially eradicated when the cells were simultaneously treated with TMZ and specific inhibitors of the ERK1/2 kinase signaling pathways [19]. In addition, the MAPK/c-Jun N-terminal kinase (JNK) signaling transduction pathway functions to induce defence mechanisms that protect organisms against various stress situations. And this pathway has also been repeatedly linked to the molecular events involved in autophagy regulation [60]. Lin CJ et al. reported that TMZ-induced autophagy was mediated by JNK activation in U87 cell lines, and the JNK inhibitor, SP600125, inhibited cell autophagy, furtherly increasing the percentage of cells undergoing apoptosis [61].

Eukaryotic elongation factor-2 kinase (eEF-2 kinase, also known as calmodulin-dependent protein kinase III), a critical enzyme controlling protein translation, is up-regulated in several types of malignancies, including gliomas [62]. Studies have reported that the expression and activity of eEF-2 kinase favor glioma cell survival and by blunting the autophagic response, eEF-2 kinase modulates the sensitivity of tumor cells to therapeutic agents, such as curcumin [63] and MK-2206 [64]. Liu XY et al. found that inhibiting eEF-2 kinase with siRNA or the inhibitor 1-Hexadecyl-2-methyl-3-(phenylmethyl)-1H-imi-dazolium iodide (NH125) could enhance the anti-glioma activity of TMZ, and this sensitizing effect was associated with the blockade of autophagy and the augmentation of apoptosis caused by TMZ [65].

## Mitochondrial electron transport chain inhibitors

Autophagy is a crucial process for cells to maintain homeostasis and survival through the degradation of cellular proteins and organelles, including mitochondria and ER [66]. Studies have indicated that TMZ could induce ROS/ERK-mediated cytoprotective autophagy to protect glioma cells from apoptosis [42, 61]. When treating the glioma cells with a combination of TMZ and mitochondrial electron transport chain inhibitors, such as rotenone, sodium azide, oligomycin or the ER stress inhibitor 4-phenylbutyrate, the TMZ-induced apoptosis and cell death could be significantly augmented by inhibiting autophagy [61].

## Vesicle-associated membrane protein 8 siRNA

Soluble N-ethylmaleimide-sensitive factor receptors (SNAREs) are a super family of small proteins with more than 35 members in mammals, varying in size and primary structure. As an essential mechanism for cellular activities, SNAREs have been observed in the progression of various tumors [67, 68]. Vesicle-associated membrane protein 8 (VAMP8), first identified as an endosomal SNARE, is significantly overexpressed in human glioma specimens and promotes cell proliferation. Furthermore, VAMP8 contributes to TMZ resistance by elevating the autophagic level, while silencing of VAMP8 using siRNA could impair the autophagic flux and alleviate TMZ resistance in glioma cells [69].

## Isocitrate dehydrogenase 1 mutation

Genetic and epigenetic studies, such as the Cancer Genome Atlas Project (TCGA), are finding enormous heterogeneity in the mutations and other genetic aberrations among GBM patients [70]. Isocitrate dehydrogenase 1 (IDH1) is a potential biomarker and drug target for GBM. Mutations of IDH1 are one of the most common and earliest detectable genetic alterations in low-grade diffuse gliomas, and evidence supports this mutation as a driver of gliomagenesis [71]. Among these mutations, the R132H mutation seems to be a more powerful prognostic marker in slow-growing gliomas, and it is associated with a more favorable outcome and better response to TMZ [72]. Gilbert MR et al. recently reported that the autophagy substrate p62/sequestosome-1 protein accumulates in both U87 cells that overexpress the R132H mutant protein and patient-derived IDH1-mutant tumors [73]. These findings suggest that the IDH1 mutation leads to the inhibition of autophagic flux, resulting in the promotion of cell death. Thus, attenuating autophagic activation may contribute to a better response to TMZ in IDH1 mutant tumors.

### Plant-derived compounds

To enhance the benefit of TMZ in the treatment of aggressive glioblastomas, effective combination strategies that sensitize glioblastoma cells to TMZ are important to prevent the recurrence of these tumors. In this regard, natural products, such as flavonoids, have received considerable attention because of their lower amount of side effects and effectively inhibition of autophagy-mediated the pro-survival roles [74]. Chrysin, the most active ingredient of pine needle extract, markedly inhibited TMZ-induced autophagy and induced apoptosis, indicating that chrysin may serve as a potential anticancer agent against glioblastomas [75]. Resveratrol (Rsv), a natural, purified polyphenolic compound, has additive toxicity with TMZ in several glioma cell lines in vitro [76] and in vivo [42]. Lin CJ et al. found that Rsv acts synergistically with TMZ in apoptosis, which is accompanied by a decrease in TMZ-induced cytoprotective autophagy. The co-administration of Rsv and TMZ reduced tumor volumes by suppressing ROS/ERK-mediated autophagy and subsequently inducing apoptosis in a mouse xenograft study [42]. Autophagy inducer sulforaphane (SFN), a isothiocyanate derived from cruciferous plants [77], could remarkably suppress cell growth and enhance cell death in TMZ-resistant glioblastoma cells and xenografts [78]. Given autophagy inhibition could enhance SFN-induced apoptosis in the breast cancer [79], prostate cancer [80], *etc.*, combination autophagy inhibitor and SFN might be a synergistically promising strategy for the TMZ treatment on GBM. Additionally, due to protective autophagy mechanism both in vitro and in vivo, the synergy between therapeutic agent curcumin and TMZ was not achieved. Autophagy inhibition could improve the efficacy of curcumin/TMZ combination therapy, providing novel opportunities to improve brain tumor treatment [81].

As the different physicochemical property of plant-derived extracts and stimulation intensity on cells, other progresses have shown that some compounds, like the *Cannabis sativa* (CS), could enhance the TMZ sensitivity by inducing the autophagic cell death. Even though most people are familiar with the palliative effects of the primary psychoactive constituent of CS, non-psychoactive cannabinoids can inhibit tumor cell viability, invasion, metastasis, and angiogenesis of cancer cells, such as glioma cell lines, which are closely related to autophagy and apoptotic-mediated cancer cell death [82, 83]. Studies have found that △^9^-tetrahydrocannabinol (THC), the main active component of CS, can induce autophagy-mediated cell death through the stimulation of endoplasmic reticulum stress or the midkine/ALK (anaplastic lymphoma kinase) axis and can further sensitize therapy-resistant tumors to antitumor action [84, 85]. Torres S et al. found that the combined administration of THC and TMZ exerts a strong anti-tumor action in glioma xenografts and TMZ-resistant xenografts with MGMT-positive T98G cells, an effect that relies, at least in part, on the stimulation of autophagy-associated cell death in tumor cells. However, the inhibition of the autophagic process using the class III PI3K inhibitor 3-methyladenine (3-MA) could prevent TMZ and THC-induced cell death [86]. Alternative attractive compound to sensitize the cells to TMZ is a steroidal lactone derived from several genera of the *Solanaceae* plant family, Withaferin A (WA). Combination treatment with WA and TMZ resulted in resensitization of MGMT mediated TMZ-resistance by Akt/mTOR pathway inhibitory modulation [87], which probably enhance the autophagic cell death in PTEN-null U87 glioma cells [88].

### Autophagy-associated cell death

In the treatment of glioblastomas, chemotherapeutic drugs, including arsenic trioxide and TMZ [89], can trigger autophagy-associated cell death and further improve their therapeutic effects. Autophagy inhibition may produce controversial cellular outcomes, including cytoprotection as alluded above and autophagy-associated cell death. Autophagy-associated cell death exerts its effect primary through the overactivity of autophagy, by which the degradation of cytoplasmic content proceeds to completion. Using siRNA against the Beclin1 or ATG7 genes totally prevents the decrease in viability after radiation/TMZ treatments in T98G and U373 glioblastoma cell lines [23]. In addition, autophagy-mediated apoptosis stimulating agents, such as Δ9-tetrahydrocannabinol [86] and oncolytic adenovirus CRAd-Surivin-pk7 [90], combined with TMZ strongly reduce the growth of glioma xenografts, suggesting that the combined administration of TMZ and autophagy inhibitors could be therapeutically exploited for the management of GBM. These results enforce the concept that autophagy-associated cell death might constitute a possible adjuvant therapeutic strategy to enhance conventional GBM treatments (Tables 2 and 4).

**Table 4:** *In vitro* studies of autophagy inducers on the TMZ anti-glioblastoma activity

Cell lines	Therapeutic methods (concentration /exprosure time)	Major findings	Interpretation	Reference
Human U87/EGFR and U251 cells	TMZ 5 and 50μM/48-72 hours dasatinib 200 nM/48-72 hours	Augmentation of Dasatinib-Induced Autophagy in combination with Temozolomide.	TKI increased autophagic cell death and sensitivity of TMZ therapy.	[93]
Human T98G and U373 cells	TMZ (300 μM) was added to the culture immediately after IR/ time: N/A rapamycin 0.1, 0.5, and 1 mM/24 hours	Autophagy-associated cell death sensiyized glioma cells to combined radiotherapy/ TMZ treatments.	Rapamycin-mediated autophagy promoted malignant glioma cell death induction after combined radiotherapy/TMZ treatments.	[23]
Human U251, U87, and T98G cells	TMZ 25 μM/24 hours IR 6Gy/6 hours PI103 0.4 μM/24 hours	A dual inhibitor of class I PI3K/mTOR, PI103, increased the cytotoxic effect of radiation therapy plus TMZ.	Enhanced radiosensitizing effects of TMZ by PI103 induced the autophagy and apoptosis, and reversed the EMT.	[100]
Human NCH82 cells	TMZ 500 μM/72 hours SKI 10 μM/72 hours	SKI could sensitize GBM cells to TMZ treatment.	Combination of TMZ and SKI resulted in autophagic flux increased and further induction of cell death potentiation.	[105]
Human T98G and SF295 cells	TMZ 25μM/96 hours VPA 1mM/96 hours	VPA increased the sensitivity of glioma cells to TMZ.	VPA enhanced the activities of TMZ on glioma cells through blocking cell cycle and promoting autophagy.	[109]
Human U87, U343, LNT-229, and MZ-54 cells	TMZ100μM/96 hours (−)-Gossypol 15μM/48 hours	Pan-Bcl-2 inhibitors augmented the action of TMZ on apoptosis-resistant malignant glioma cells.	Pan-Bcl-2 inhibitors (−)-Gossypol induced caspase-independent, autophagic cell death when combined treatment with TMZ.	[112]
Human T98G and U373 cells	TMZ 100μM/48 hours EGFR SiRNA 1μM/72 hours	EGFR interfering resulted in an increase of TMZ cytotoxicity in TMZ-resistant GBM cells.	EGFR SiRNA inhibited the pro-death autophagy and sensitized GBM cells to subsequent TMZ treatments	[97]
Human U251 cells	TMZ 100 μM/72 hours Nrf2 shRNA N/A	Combination of TMZ and the knockdown of Nrf2 could enhance the antitumor effects of TMZ in GBM.	Knockdown of Nrf2 by shRNA enhanced autophagy induced by TMZ.	[117]
Human U87, T98G, and HG19 cells	TMZ 25-75μM/72 hours THC 0.9μM/72 hours	Coadministration of TMZ with THC exerted a strong antitumoral action in glioma cells.	Combined administration of THC and TMZ enhanced autophagy-mediated apoptosis in tumor cells.	[86]
Human T98G and U251 cells	TMZ 300-500 μM/24 hours WA 0.5-2μM/24 hours	Combination treatment with WA and TMZ resulted in resensitization of TMZ-resistance	Withaferin A resensitizes TMZ-resistant GBM cells to TMZ through MGMT depletion	[87]
Human U87 and U373 cells	TMZ 100 μM/24 hours oncolytic adenovirus 100 vp per cell/24 hours	Oncolytic adenovirus led to improved efficacy of TMZ treatment against a panel of glioma cell lines.	Combination of oncolytic adenovirus with TMZ increased tumor cell autophagy and apoptosis-mediated cell death.	[90]
Rat RG2 cells	TMZ 100 μM/48 hours PTx 20 ng/ml /48 hours	PTx has the potential to be useful as an adjunct to TMZ chemotherapy on glioma.	Concomitant treatment with TMZ and PTx elicited autophagic cell death in vitro and increased the survival in RG2 glioma model.	[122]

## Kinase inhibitors

Though some kinases inhibitors above mentioned have been proved to increase the cytotoxicity of TMZ by inhibiting the cell autophagy, recent studies have indicated that other kinase inhibitors, like the tyrosine kinase inhibitors (TKI), could cause the remarkable autophagic cell death [91], and resulted in a significant reduction in glioma tumor growth [92]. Milano V’s group found that Dasatinib (BMS-354825), an orally bioavailable tyrosine kinase inhibitor, could lead to a significant increase in the sensitivity to TMZ therapy via generating cell cycle disruption and autophagic cell death [93]. Furthermore, the cell surface receptor, epidermal growth factor receptor tyrosine kinase (EGFR-TK) is highly amplified, mutated, and overexpressed in human malignant gliomas [94]. EGFR signaling could induce the phosphorylation of pro-survival STAT3, ERK1/2 and Akt, which contributes significantly to GBM cell proliferation [95]. Thus, therapeutic strategies to inhibit EGFR kinase activity represent an avenue of profound beneficial effects for gliomas. The combined treatment of nimotuzumab (monoclonal antibody against EGFR) and rapamycin effectively enhances glioma cell death in TMZ-resistant glioma cells [31]. The over-expression of miR-340 suppressed several oncogenes, including EGFR, and further dramatically inhibited glioma cell proliferation, induced cell-cycle arrest and apoptosis, and promoted autophagy [96]. EGFR interference using siRNA results in an increase of TMZ cytotoxicity in T98G TMZ-resistant cells, which was through activation of a pro-death autophagy process [97].

The aberrant PI3K/Akt/mTOR pathway has been shown to contribute to the resistant phenotype of gliomas [36, 98]. Therefore, the PI3K/Akt/mTOR pathway is regarded as an important amenable pathway for pharmacological interventions in gliomas. In radioresistant glioma cells, treatment with the mTOR inhibitors rapamycin and PP242 can enhance radiosensitivity by potently and persistently activating the autophagic flux [99]. After combined radiotherapy and TMZ treatments, rapamycin-mediated autophagy is able to promote malignant glioma cell death [23]. Another group also found that PI103, a dual inhibitor of PI3K and mTOR, could increase autophagy and further increase the cytotoxicity of radiation and TMZ [100]. However, it was note worthy that, as opposed to the previous findings, treatment with rapamycin alone did not discernibly potentiate the radiosensitizing effect of TMZ in both U251 and T98G cells [100]. These results suggest that more careful studies are needed to determine optimal treatment combinations of TMZ and mTOR inhibitors.

Sphingolipids are structural and functional components of biological membranes, which benefit the maintenance of membrane structure and fluidity. They are also implicated in bio-effector roles in cancer pathogenesis. The roles of bioactive sphingolipids, specifically sphingosine kinase 1 (SK1) and 2 (SK2) and their product—sphingosine 1-phosphate (S1P), have been shown to regulate the cancer cell proliferation, survival, and treatment responses. Modulating the metabolism of bioactive sphingolipids has been shown to be a potentially important target in treating malignancies [101, 102]. Particularly, because sphingosine kinases (SK1 and SK2), serving as the oncogenic enzymes, have been found to induce transforming phenotype in many tumors, including glioblastomas [37, 103], inhibition of SK may become a promising anticancer strategies [104]. Noack J et al. found that the combination of TMZ and sphingosine kinases inhibitors (SKIs) resulted in an increase autophagic flux and further induced cell death in GBM cell lines. This role of autophagy-associated cell death in the combination of SKI and TMZ treatment was demonstrated by the decrease in cell death after specific and efficient the siRNA-mediated knockdown of Beclin1 [105].

## Histone deacetylases inhibitors

Histone deacetylases (HDACs) constitute a family of enzymes that play important roles in the epigenetic regulation of gene expression and contribute to the growth, differentiation and apoptosis of cancer cells, including glioblastomas [106]. Recently, strategies to enhance tumor cytotoxicity and radiosensitivity have started to focus on HDACs. Many HDAC inhibitors have been demonstrated to enhance the cytotoxicity and therapy sensitivity of human glioma cell lines [107]. Among these HDAC inhibitors, 2-propylpentanoic acid (VPA) is one of the most interesting. VPA is a short-chain fatty acid that belongs to the HDAC inhibitor family. A combination of VPA and TMZ has a significantly enhanced antitumor effect in TMZ-resistant malignant glioma cells. This enhanced antitumor effect correlates with enhanced apoptotic and autophagic cell death [108]. Chen’s group also found if combined with VPA for 96 hours, the sensitivity of glioma cells to TMZ was significant increased. The combination treatment of TMZ and VPA results in a significant cell cycle block and increased apoptotic rates as well as autophagy rates in T98G and SF295 cell lines [109].

## Bcl2 family inhibitors

As mentioned earlier, antiapoptotic Bcl-2 family members, such as Bcl2L12, suppress both apoptosis and autophagy, and they are of major importance for therapy resistance of malignant gliomas [110]. The deregulation of Bcl2 family proteins mostly contributes to apoptosis evasion, suggesting that the inhibition of Bcl2 proteins is one of the most promising new approaches to targeted cancer therapy [111]. The Bcl2 inhibitor ABT-737 could counteract the anti-apoptotic role of Bcl2L12 and sensitize drug response of GBM cells to TMZ [110]. In addition, the pan-Bcl2 inhibitor (−)-gossypol efficiently potentiates caspase-independent autophagic cell death in apoptosis-resistant malignant glioma cells, and it further augments the action of TMZ. The extent of this cell death could be strongly diminished by the lentiviral knockdown of Beclin1 and ATG5 [112].

## Nuclear factor E2-related factor 2 inhibition

Nuclear factor E2-related factor 2 (Nrf2), a pivotal transcriptional factor of cellular responses to oxidative stress, is observed to function remarkably in glioblastoma pathobiology. Nrf2 activation contributes the tumorigenesis of autophagy-deficient cells [113]. In addition, a significant negative correlation has been found between Nrf2 expression and the outcome for GBM patients [114]. Recent studies have reported that development of chemoresistance is associated with the constitutive activation of the Nrf2-mediated signaling pathway in many types of cancer cells, including gliomas [115]. Chrysin, a potent Nrf2 inhibitor, could effectively reverse the resistance of an anticancer drug by down-regulating the PI3K/Akt and ERK pathways [116]. The knockdown of Nrf2 by siRNA enhances autophagy induced by TMZ and decreases the viability of U251 cells [117]. These findings suggest that the combination of TMZ and the inhibition of Nrf2 may point to a novel therapeutic opportunity for GBM to enhance the antitumor effects of TMZ.

## Oncolytic adenoviruses

The potential use of adenoviruses in therapy against cancer has evoked a rapidly moving field of research. Emerging evidence indicates that as a cancer drug, oncolytic adenoviruses can induce autophagic cell death in glioma cancer cells [118]. The use of autophagy inducers, such as rapamycin, can enhance the oncolytic potency of recombinant adenoviruses. Furthermore, studies have demonstrated the capability of adenoviruses to inhibit the expression of the DNA repair enzyme MGMT and to chemosensitize glioma cells to TMZ [119]. As oncolytic adenoviruses show promising safety and efficacy, the combination of oncolytic adenoviruses with TMZ could increase tumor cell autophagy and elicit antitumor immune responses, resulting in disease control in 67 % of chemotherapy refractory cancer patients [120]. Ulasov IV et al. also found that pretreatment with TMZ, followed by treatment with oncolytic adenovirus CRAd-Surivin-pk7, exhibits an additive cytotoxicity effect in vitro and in vivo, which is associated with increased autophagy-associated cell death and a therapeutic additive effect in the survival of mice bearing intracranial glioma xenografts [90].

## Pertussis toxin

Pertussis toxin (PTx), an exotoxin produced by *Bordetella pertussis*, regulates the activation induced by autophagic process in cancer cells [121]. A recent study indicated that PTx has the potential to be useful as an adjunct to TMZ chemotherapy on gliomas. Concomitant treatment with TMZ and PTx can elicit autophagic cell death *in vitro* and increase survival in the RG2 glioma model [122].

## Conclusions

Resistance to TMZ chemotherapy is a major obstacle to the success of glioma therapy. The roles of autophagy regulation, which cause multiple impacts on chemosensitivity, are still highly perplexing in glioma treatment. Clearly, the regulation of autophagy corresponding to TMZ therapy and the resulting downstream effects are complex and are very likely to be in a cell type-specific manner. The competence of a cell to survive or die is theoretically proportional to the doses and duration of TMZ treatment, the DNA-damage repair capacity of the cells, the proliferation level, and the effectiveness of activating DNA repair proteins including ATM kinases. How can the autophagy act as the pro-survival or pro-death roles, and how are the decisions made? Hypothetical models predict that with the low doses and short-term TMZ treatment, autophagy is a survival mechanism, whereas upon the persistent TMZ treatment, autophagy becomes a process that is out of control and induce the cell death. With this context, the treatment thresholds have an import role. These pro-death and pro-survival pathways, and how they interact, are needed to be discussed more in the future.

Although the controversy about the prosurvival or anticancer effect of autophagy is still heated, the data in clinical trials seem to support the cytoprotective role of autophagy inhibitors, such as CQ and its analogs, preceding treatment with TMZ. However, whether other different autophagy inhibitors, such as bafilomycin A1, monensin, 3-MA, pyrvinium and wortmannin, which block the autophagic process at different stages, have the same pharmacological features as CQ are still unknown. At the present, it is not completely clear how autophagy influences cells, especially in TMZ resistant tumors. Moreover, it remains unclear how autophagy participates in the unique mechanical properties of microenvironments (e.g., hypoxia and acidity) under TMZ exposure. Further investigation of these issues may help to identify more combination strategies to enhance the benefits of TMZ chemosensitivity and chemoprotection in the treatment of aggressive glioblastomas.

## Abbreviations

GBM: glioblastoma multiforme
TMZ: temozolomide
ROS: reactive oxygen species
MGMT: O^6^-methylguanine-DNA methyltransferase
ATM: Ataxia-telangiectasia mutated
ERK: extracellular signal-regulated kinase
AMPK: adenosine monophosphate-activated protein kinase
eEF-2: eukaryotic elongation factor-2
VAMP8: vesicle-associated membrane protein 8
CQ: Chloroquine
mTOR: the mechanistic target of rapamycin (serine/threonine kinase)
SKI: sphingosine kinases inhibitor
EGFR: epidermal growth factor receptor
Nrf2: nuclear factor E2-related factor 2
